# The spatiotemporal evolution of toponymic cultural landscapes in Hebei Province, China, and its influencing factors

**DOI:** 10.1371/journal.pone.0353417

**Published:** 2026-07-20

**Authors:** Da Zhang, Han Song, Pingzhi Li, Yixi Guo, Yating Luo, Sheng Mao, Zijia Feng, Shuwen Zheng, Zhuoyan Jiang

**Affiliations:** 1 School of Environment and Disaster Management, University of Emergency Management, Beijing, China; 2 Hebei Key Laboratory of Resource and Environmental Disaster Mechanism and Risk Monitoring, Sanhe, China; Bahir Dar University, ETHIOPIA

## Abstract

Current research on toponymic cultural landscapes lacks systematic attention to geographical transition zones and often neglects the interpretation of long-term evolutionary processes and driving mechanisms. This study examines 1,854 township-level place names in Hebei Province, China, by integrating methods such as kernel density estimation, geographic detectors, and spatial autocorrelation analysis. Focusing on the transition zone along the Taihang Mountains–Great Wall corridor, patterns of spatiotemporal differentiation and the dual mechanisms shaping changes in place names are investigated. The findings reveal significant spatiotemporal variation in Hebei’s place names. Natural environment names have contracted toward northeastern mountainous and coastal regions, while cultural names have shifted northward. The transition zone exhibited a “military-to-civilian” transformation characteristic during the Ming and Qing dynasties. Slope gradient and vegetation coverage are identified as primary natural drivers, whereas ethnic integration and migration during the Ming Dynasty constitute the key social factors. This study enriches interdisciplinary research on human–land relationships and toponymic landscapes in transition zones, proposes region-specific conservation strategies, and provides a scientific basis for the protection of toponymic cultural heritage in similar transition zones across Hebei and North China.

## Introduction

Place names are composite symbols that integrate geographical spatial identification with historical and cultural attributes. They embody regional cultural contexts and human–environment interactions while concealing the underlying logic of local identity, social power, and collective memory [1]. Under the combined influence of globalization, rapid urbanization, and weakening awareness of historical and cultural heritage, the integrity of toponymic cultural landscapes continues to be compromised [2], making systematic protection imperative. Interpreting regional human–environment relationships from the perspective of toponymic evolution holds significant theoretical and practical importance for both the preservation of toponymic cultural heritage and sustainable development.

The international academic community has long engaged in the study of toponymic culture, with existing research primarily focusing on three core themes. The first concerns the linguistic and cognitive attributes of place names and their cultural connotations. This line of research emphasizes the analysis of etymology, structure, semantics, and cognitive patterns, revealing underlying linguistic systems and cultural meanings through semantic classification and etymological tracing [3]. By employing methods such as cognitive linguistics, semantic analysis, and cross-cultural comparison, scholars explore the symbolic meanings of place names, integrate multi-source data, and analyze the grammatical and phonological features of multilingual toponyms. These approaches demonstrate that place names function both as linguistic symbols and as concrete carriers of regional culture and cognitive processes, thereby clarifying the intrinsic relationship between linguistic characteristics and cultural connotations [4–7].

The second theme focuses on the social functions and power dynamics of toponyms, highlighting their roles in social memory, identity construction, and the expression of power. Researchers commonly employ questionnaires, interviews, and archival analysis to examine the historical stratification and collective memory embedded in place names [8], or adopt multidisciplinary theoretical frameworks to analyze the political struggles and ethnic relations underlying toponymic change [9,10]. In particular, research on indigenous place names emphasizes their political and cultural implications [11], while critical toponymy reveals the symbolic and practical functions of place names in political and economic contexts [12,13].

The third theme addresses the spatial patterns and evolutionary mechanisms of place names, with a focus on spatial differentiation and driving factors. Technologies such as geographic information systems (GIS), kernel density analysis, and geographic detectors are widely used to identify the spatial distribution patterns of natural and cultural place names and their relationships with topography, geomorphology, and population migration [14]. Quantitative analyses based on regional databases and historical documents have effectively identified dominant factors influencing naming patterns [15], while cross-regional comparisons and mixed qualitative–quantitative approaches have further demonstrated their advantages in such research [16,17].

Despite substantial research progress, several limitations remain. First, spatiotemporal coupling analyses of toponymic cultural landscapes remain inadequate [18]. Many studies emphasize static interpretations of linguistic structures or power symbols, lacking long-term, multidimensional analyses that treat place names as dynamic cultural landscape units. Second, there is an imbalance in the research scale. Existing studies have largely focused on provincial, municipal, or specific typological levels, with insufficient consideration of geographical contexts [19]. In particular, limited attention has been paid to toponymic landscapes at the township scale, which embody rich regional cultures, as well as to those located in natural geographical transition zones and their evolutionary patterns.

This study selects Hebei Province as the case study area. Hebei Province is situated in the northern agricultural region of China and has long served as a vital imperial hinterland across successive dynasties [20]. Characterized by a transitional climate and diverse landforms [21], the region exhibits typical characteristics of toponymic evolution shaped by the combined influences of natural conditions, migration, military activities, and related factors. [22] Existing studies are largely confined to specific localities [23] or single types of toponyms [24], and lack systematic spatiotemporal coupling analyses at the provincial scale [25,26].

Accordingly, taking township-level toponyms in Hebei as the research subject, this study constructs a cultural classification system, integrates spatiotemporal evolution analysis with the Geodetector model, and focuses on topographic and climatic transition zones. The dominant role of natural factors is quantitatively assessed, the spatiotemporal differentiation characteristics of toponyms and their driving mechanisms are revealed, and the social dimensions of toponymic evolution, including ethnic migration, military operations, and economic development, are clarified. This study aims to enrich the theoretical framework of toponymic cultural landscapes in transition zones and to provide a scientific basis for the protection of toponymic cultural heritage and the advancement of local cultural construction.

## Methodology

### Overview of the study area

**Fig 1:**
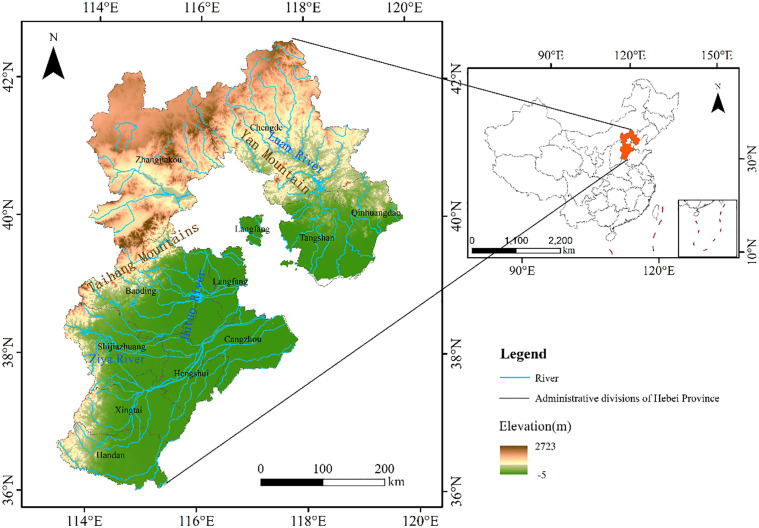
The overview of the study area in Hebei Province. Note: This figure was created using map data from Tianditu, available at http://www.tianditu.gov.cn/.

Township place names refer to the official names of grassroots administrative units in China, including townships, towns, and ethnic townships, as well as commonly used names formed through historical evolution [27]. Town typically denotes a grassroots settlement with relatively high urbanization levels and concentrated populations, while ‘township’ focuses more on agricultural regions. In this study, ‘township place names’ refer to the official names of basic administrative units such as China townships, towns, and ethnic townships, as well as the commonly used names formed during historical evolution.As composite geographical entities integrating natural features, historical–cultural accumulation, and social change, township place names function not only as spatial identifiers but also as carriers of rich information on regional cultural heritage and the evolution of human–land relationships. Hebei Province is located in the central part of the North China Plain, extending from 36°05′ to 42°40′ N and from 113°27′ to 119°50′ E (Fig 1). It borders the Bohai Sea to the east, the Taihang Mountains to the west, and the Yan Mountains to the north, and is adjacent to the Bashang Plateau in the northwest, as well as Henan and Shandong Provinces to the south. The province surrounds Beijing and Tianjin, forming the core area of the Beijing–Tianjin–Hebei urban agglomeration. The overall terrain slopes from west to east and features complex and diverse landforms, forming a typical transition from mountainous and plateau regions to plains [25]. Hydrologically, Hebei Province lies within the Haihe River Basin and is characterized by well-developed water systems and dense river networks [28]. Ecologically, mountainous areas are dominated by deciduous broad-leaved forests [29], while plains are primarily covered by extensive farmland [30]. Vegetation types exhibit clear gradient distributions influenced by altitude, terrain, and climate. In addition, the 400-mm annual precipitation isohyet runs through the central part of the province, marking the transitional boundary between arid–semiarid and semi-humid zones [31]. These natural geographical elements intersect spatially to form agro-pastoral transition zones, climatic transition zones, and geomorphic step zones, providing a diverse and dynamic natural foundation for the formation and evolution of place names.

From a cultural and historical perspective, Hebei is one of the important cradles of Chinese civilization. The Nihewan site preserves early evidence of human activity [32], while the Cishan culture represents the development of primitive agricultural civilization during the Neolithic period [33]. During the Spring and Autumn and Warring States periods, the region was governed by the states of Yan and Zhao, laying the foundation for its diverse regional culture [34]. In the feudal era, particularly during the Yuan, Ming, and Qing dynasties, Hebei served as a key area surrounding the imperial capital and the core of the northern military defense system. During this period, frequent historical processes such as population migration, military garrison farming, and administrative reorganization significantly enriched the socio-cultural connotations and historical layers of the province’s toponymic system [35].

### Data sources

#### Geographical data and preprocessing.

This study systematically examines the naming origins and temporal evolution of township-level place names in Hebei Province, using the *Hebei Provincial Gazetteer of Place Names* [36] as the primary reference to establish a standardized attribute database. To determine the age of place names, the earliest recorded appearance in the *Hebei Provincial Gazetteer of Place Names* [36] and in local gazetteers (e.g., county-level records) was used as the principal criterion. For townships that have undergone renaming, the earliest established name was adopted as the statistical benchmark. Place names with missing records or ambiguous dates were classified as “undetermined” and excluded from subsequent chronological analysis to ensure data reliability. To improve the timeliness and accuracy of the dataset, administrative boundary adjustments and toponymic changes since 2007 were comprehensively verified through official sources, including the website of the Ministry of Civil Affairs of the People’s Republic of China (https://www.mca.gov.cn), Guangming Net (https://www.gmw.cn), Duxiu Academic Search (https://www.duxiu.com), and other administrative division platforms. The original dataset was then rigorously cleaned and validated, resulting in 1,854 valid samples of township place names. Specifically, 1,518 (81.9%) samples are sourced from official place name documents such as the Hebei Provincial Gazetteer of Place Names [36] and county-level local records, and the remaining 336 (18.1%) samples are supplemented from the above official online channels.

Based on existing studies by scholars such as Lu et al. [37] and Zhang et al. [26], as well as general toponymic classification standards, place names were categorized into two primary types: natural landscape place names and cultural landscape place names. The natural landscape category includes 501 place names, further subdivided into three secondary types: those based on geomorphological features, hydrological features, and flora and fauna. The cultural landscape category comprises 1,345 place names, covering seven types: surnames, those based on military affairs, economic activities, architecture, transportation, religion, and directional markers. In addition, eight place names were classified as “other” due to their unique attributes.

To balance historical continuity with phased evolutionary characteristics, the study period was divided into four stages: before the Yuan Dynasty, the Yuan Dynasty, the Ming Dynasty, and from the Qing Dynasty to the modern era. Statistical results indicate that 449 place names originated before the Yuan Dynasty, 66 during the Yuan Dynasty, 570 during the Ming Dynasty, and 410 from the Qing Dynasty to the modern era, while 359 place names lack clear formation dates. The quantitative distribution and structural characteristics of each category are presented in Table 1.

**Table 1:** The statistics of township place names in Hebei Province.

Primary type	Number of place names (units)	Proportion (%)	Secondary type	Number of place names (units)	Proportion (%)
Natural landscape	501	27	Geomorphology	167	9.0
			Hydrology	227	12.3
			Flora and fauna	107	5.8
Cultural landscape	1345	72.6	Surnames	473	25.5
			Military affairs	163	8.8
			Economic activities	141	7.6
			Architecture	40	2.2
			Transportation	192	10.4
			Religion	193	10.4
			Direction	143	7.7
Other	8	0.4	Not classified	8	0.4
Total	1854	100		1854	100

#### Influencing factor data.

To investigate the factors influencing toponymic evolution, a database integrating both natural and cultural attributes was developed. The dots and polygons data of administrative divisions of China at a scale were obtained from National Catalogue Service For Geographic Information (http://www.tianditu.gov.cn/). Natural environmental indicators included digital elevation model (DEM) elevation, hydrographic networks, and vegetation coverage, while cultural elements included vector data on migration routes of immigrants in Hebei Province, ethnic settlements (autonomous counties and townships), ancient military defense systems, and the spatial distribution of the Great Wall. Detailed attributes and sources of each dataset are presented in Table 2.

**Table 2:** The statistical summary of influencing factors.

Data classification	Data type	Data sources	Data usage
Natural environment data	30-m DEM topographic data	Yamazaki Lab website (https://hydro.iis.u-tokyo.ac.jp/~yamadai/)	Analysis of the correlation between natural environment place names and topography, water systems, and vegetation cover
	Hebei Province waterway data	BBBike Extract Service (https://extract.bbbike.org/)	
	Monthly vegetation coverage data (250-m resolution)	National Tibetan Plateau Science Data Center Platform (https://data.tpcd.ac.cn/)	
	Taihang Mountains boundary	Institute of Geographic Sciences and Natural Resources Research, Chinese Academy of Sciences (https://www.resdc.cn/)	Analysis of the evolution of place names in terrain transition zones
Cultural data	Immigration data	*China Immigration History*, Vol. 5: Ming Dynasty [38]; *A Historical Walk in Huainan 4* [39]	Analysis of the correlation between cultural place names and population migration factors
	Ethnic minority data in autonomous townships and counties	Ministry of Civil Affairs of the People’s Republic of China (https://www.mca.gov.cn/)	Analysis of the correlation between cultural place names and ethnic factors
	Ancient military geography vector data	Waterways Commentary GIS Platform (https://www.rivermap.cn)	Analysis of the correlation between cultural place names and military factors
	Great Wall of China data	Great Wall of China Platform (http://www.greatwallheritage.cn); National Geographic Information Public Service Platform (https://zrzy.tianditu.gov.cn/)	
	The Beijing–Hangzhou Grand Canal	www.openstreetmap.net.cn	

### Research methods

#### Kernel density analysis.

Kernel density estimation uses a distance-decay function to identify local density differences among spatial elements. The calculation formula is as follows:


$$\begin{array}{c} f\left(x\right)=\frac{\text{1}}{nh}\sum_{i=\text{1}}^{n}k\left(\frac{x-x_{i}}{h}\right), \end{array}$$ (1)

where f(x) represents the kernel density value; n denotes the number of points within the bandwidth; k is the kernel function; and $x-x_{i}$ is the distance between the estimation point and the sample point. The bandwidth strongly influences the smoothness of the results, and testing multiple bandwidth values is recommended [37]. In this study, kernel density analysis is primarily used for visual quantitative analysis.

#### Geodetector.

Geodetectors are used to evaluate the explanatory power of influencing factors on the spatial differentiation of the dependent variable *Y*, as expressed by the following equations [37]:


$$\begin{array}{c} q\;=\;\text{1}-\frac{\sum_{h=\text{1}}^{L}N_{h}\sigma_{h}^{\text{2}}}{N\sigma^{\text{2}}}\;=\;\text{1}-\frac{SSW}{SST}, \end{array}$$ (2)

$$\begin{array}{c} SSW\;=\sum_{h=\text{1}}^{L}N_{h}\sigma_{h}^{\text{2}}, \end{array}$$ (3)

$$\begin{array}{c} SST\;=\;N\sigma^{\text{2}}, \end{array}$$ (4)

where L represents the stratification of variable *Y* or factor *X*. The number of units in layer h and the total number of units in the study area are denoted by $N_{\text{h}}$ and N, respectively. The variances *Y* of in layer h and in the entire region are represented by $\sigma_{\text{h}}^{\text{2}}$ and $\sigma_{N}^{\text{2}}$, respectively. SSW and SST denote the within-layer variance sum and the total regional variance, respectively [40].

In this study, ArcGIS 10.8 was used to construct a 5 × 5 grid across the study area. Continuous variables, including elevation, slope, distance to rivers, and vegetation coverage, were discretized using the reclassification tool. Kernel density values of place names and corresponding influencing factor values were extracted at grid center points. After noise reduction, the data were processed using the Geodetector model for factor detection.

#### Spatial autocorrelation analysis.

Global spatial autocorrelation analysis was employed to examine the spatial correlation of township place names within the study area. The Moran’s *I* value ranges from –1 to +1, where a value greater than zero indicates a positive spatial correlation, a negative value indicates a negative correlation, and zero indicates no spatial correlation. The calculation formula is as follows:


$$\begin{array}{c} \text{Mora}{\text{n}}^{\prime }\text{s }I\;=\;\frac{\sum_{i=\text{1}}^{n}\sum_{j=\text{1}}^{n}w_{ij}\left(x_{i}-\overset{―}{x}\right)\left(x_{j}-\overset{―}{x}\right)}{\left(\sum_{i=\text{1}}^{n}\sum_{j=\text{1}}^{n}w_{ij}\right)\sum_{i=\text{1}}^{n}{\left(x_{i}-\overset{―}{x}\right)}^{\text{2}}}\left(i\;\neq \;j\right), \end{array}$$ (5)

where $x_{i}$ and $x_{j}$ represent the attribute values of the units at locations i and j, respectively; $\bar{x}$ denotes the mean of the attribute values; $w_{ij}$ is the spatial weight based on adjacency or distance, and n is the total number of units [41].

## Result analysis

### Spatial differentiation characteristics of township place names

#### Spatial distribution of natural environment place names.

**Fig 2:**
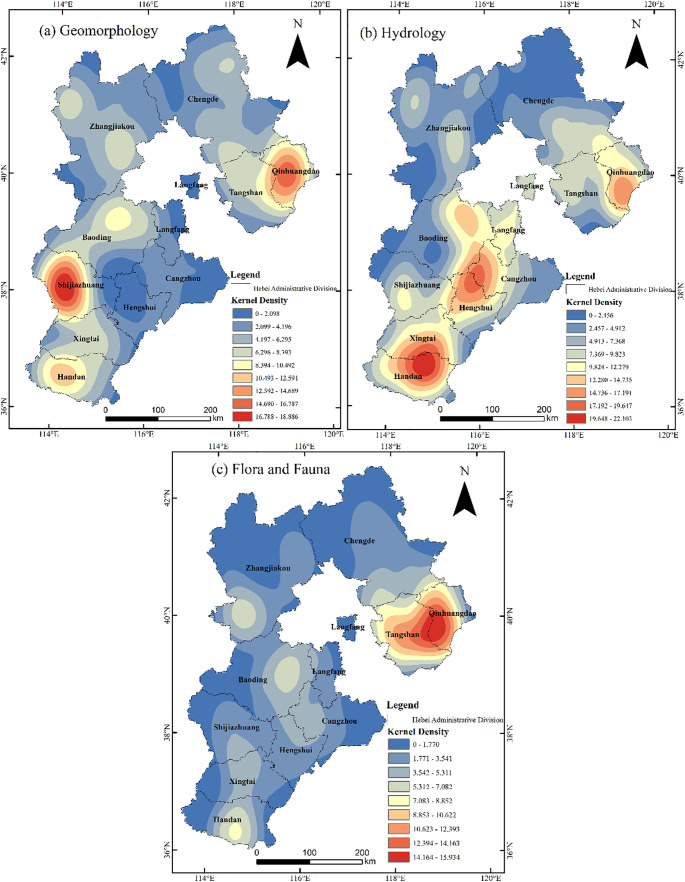
The kernel density maps of natural environment place names. Note: This figure was created using map data from Tianditu, available at http://www.tianditu.gov.cn/.

As shown in Fig 2, high-density zones of geomorphological place names are mainly located in Shijiazhuang in southwestern Hebei and Qinhuangdao in the east. Hydrological place names are concentrated in the central and southern plains, forming a continuous belt-like distribution with pronounced north–south variation. Flora and fauna place names are clustered primarily in Qinhuangdao and Tangshan in the east, with secondary clusters appearing in southern regions.

#### Spatial distribution of cultural place names.

**Fig 3:**
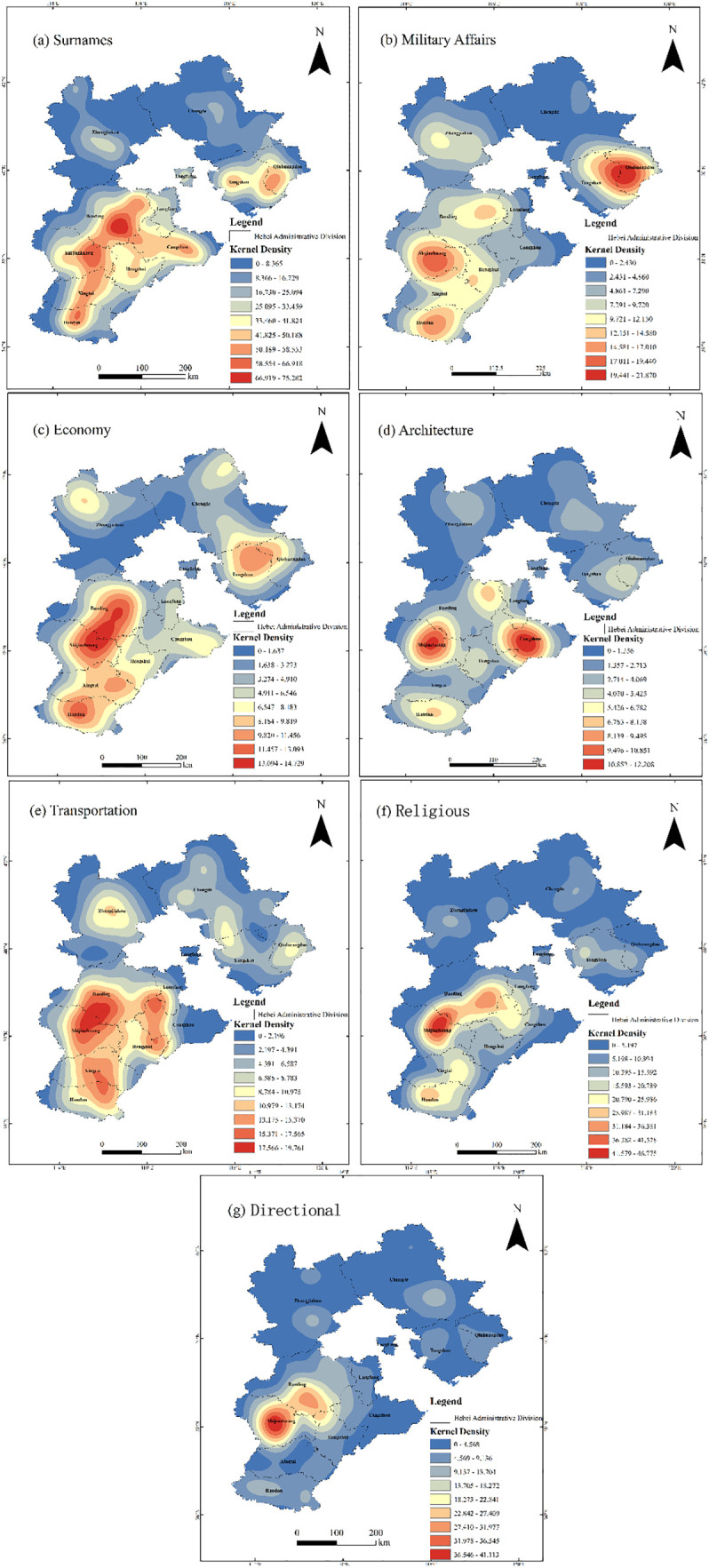
The kernel density maps of cultural place names. Note: This figure was created using map data from Tianditu, available at http://www.tianditu.gov.cn/.

As illustrated in Fig 3, different categories of cultural place names exhibit distinct spatial distributions. Surname-based names are predominantly distributed in low-altitude regions of central, southern, and eastern Hebei. Military-related names are mainly distributed across eastern areas and in the alluvial plains at the eastern foothills of the Taihang Mountains, covering extensive spatial regions. Economic activity-related names cluster around Shijiazhuang and Baoding, with secondary clusters in southern and eastern areas. Architectural place names are concentrated around Shijiazhuang and Cangzhou. Transportation- and religion-related names are mostly distributed in central–southern regions, while directional names are notably concentrated in central areas.

### Temporal evolution of township place names in Hebei Province

#### Temporal evolution of natural environment place names.

**Fig 4:**
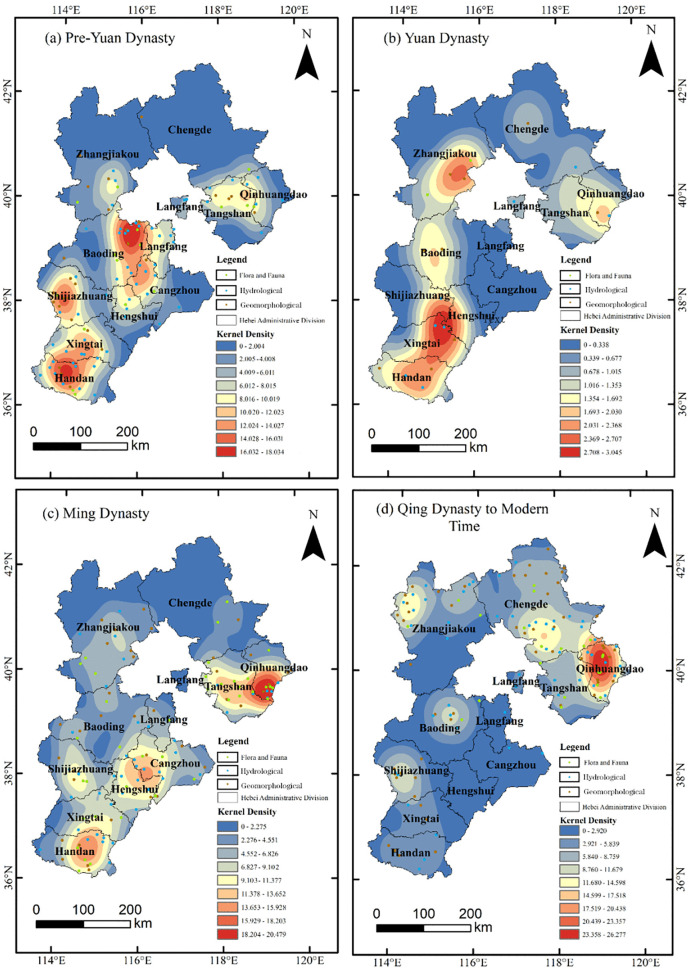
The kernel density maps of the temporal evolution of natural environment place names in Hebei townships across dynasties. Note: This figure was created using map data from Tianditu, available at http://www.tianditu.gov.cn/.

An analysis of Fig 4 indicates that prior to the Yuan Dynasty, hydrological place names dominated the natural environment category, primarily concentrated in Baoding, Shijiazhuang, and Handan. Hydrological and flora and fauna place names were widely distributed across the central–southern plains and eastern regions, while geomorphological names were highly concentrated in pre-mountain transition zones and undulating terrain along the eastern foothills of the Taihang Mountains.

During the Yuan Dynasty, the total number of natural environment place names decreased significantly, particularly in hydrological and geomorphological categories. High-density areas shifted northward to southern Zhangjiakou. Geomorphological names clustered east of the Taihang Mountains and near Zhangjiakou, hydrological names were mainly distributed in southern and northeastern flatlands, and flora and fauna names persisted in northwestern Hebei.

The Ming Dynasty witnessed a resurgence in natural environment place names, with spatial distribution expanding to form multi-core, high-density clusters centered on Tangshan, Qinhuangdao, Cangzhou, Shijiazhuang, and Handan. Hydrological and geomorphological names exhibited extensive distribution and strong spatial correlation, while flora and fauna names co-occurred in these regions.

From the Qing Dynasty to the modern era, natural environment place names declined again, especially in the flora and fauna category. High-density cores shifted northeastward, forming a primary core around Qinhuangdao and a secondary core in southern Chengde. Hydrological and geomorphological names became concentrated in high-altitude areas of the northeast, northwest, and north, whereas plains exhibited sparse distributions of natural environment place names.

In summary, the evolution of natural environment place names in Hebei Province progressed from early multi-center agglomeration in the plains, to northward expansion during the Yuan Dynasty, to wide-area, multi-core diffusion in the Ming Dynasty, and finally to concentrated clusters in northeast mountainous and coastal areas since the Qing Dynasty.

#### Temporal evolution of cultural place names.

**Fig 5:**
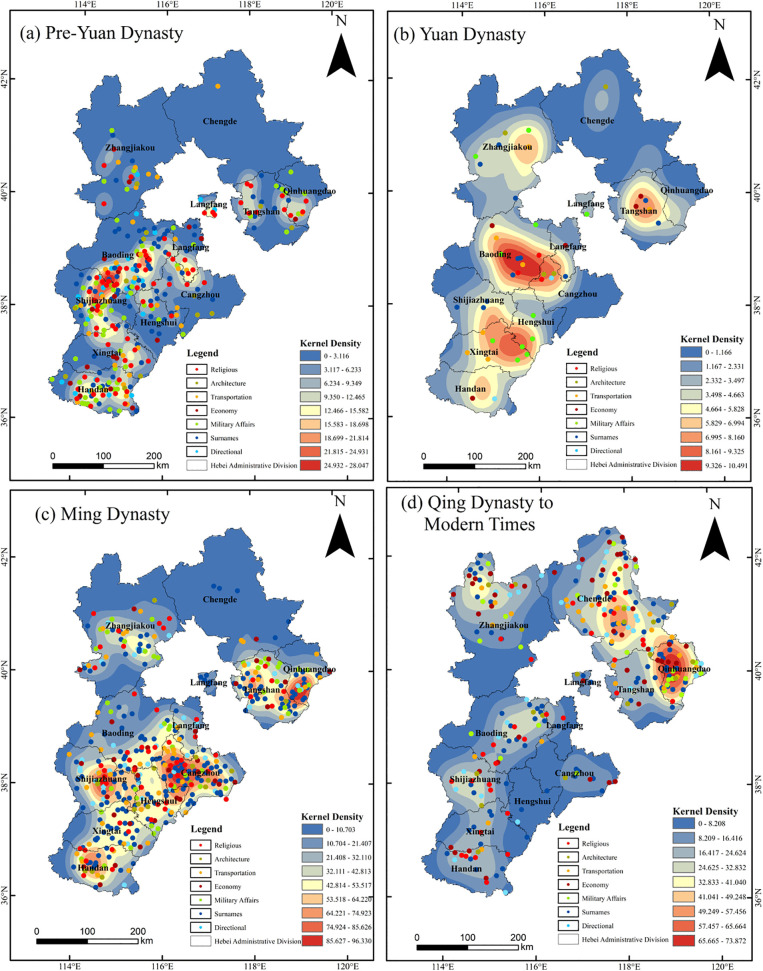
The kernel density maps of the temporal evolution of cultural place names in Hebei townships across dynasties. Note: This figure was created using map data from Tianditu, available at http://www.tianditu.gov.cn/.

As shown in Fig 5, pre-Yuan Dynasty cultural place names were mainly concentrated in Shijiazhuang, Baoding, and Handan. Categories including religious, architectural, economic, military, surname-based, and directional names were already widespread. During the Yuan Dynasty, the distribution center shifted eastward, forming a high-density zone. During the Ming Dynasty, the number of place names increased significantly, with Cangzhou, Shijiazhuang, Handan, Tangshan, and Qinhuangdao emerging as core clusters. Religious, architectural, transportation, economic, and surname-based names expanded in both quantity and spatial coverage. From the Qing Dynasty to the modern era, the distribution center shifted northeastward, concentrating in southern Qinhuangdao and Chengde. Zhangjiakou maintained a secondary density but shifted northward, while southern Hebei experienced a marked decrease in density. Overall, cultural place names in Hebei evolved spatially from central–southern plains to northern mountainous plateaus, and from inland alluvial fan plains to coastal regions.

#### Spatiotemporal evolution of place names in transition zones.

The Taihang Mountains [42] and the Ming Dynasty Great Wall [43], as typical transition zones of terrain and precipitation in Hebei, respectively illustrate the morphological gradient from plains to mountains and the ecological boundary between arid and humid climates. These zones serve as natural geographic markers and have historically formed areas of cultural tension between farming and nomadic activities, as well as between the Central Plains and frontier regions [25]. They represent typical composite transition zones in Hebei’s regional spatial structure.

Using the Taihang Mountains and the Ming Dynasty Great Wall as boundaries, the spatial distribution and quantities of toponym types on both sides are analyzed to reveal the coupling relationship between natural geographic boundaries and human spatial patterns (see Table 3).

**Table 3:** The temporal evolution of place names on both sides of transition zones.

Transition zone type	Zone	Dynasty	Total	Semantic composition of place names (quantity)
Terrain transition zone	Plain area (right)	Before the Yuan Dynasty	37	Religion (7), Direction (6), Geomorphology (5), Military affairs (5), Transportation (5), Hydrology (3), Surnames (3), Economic activities (2), Architecture (1)
		Yuan Dynasty	2	Geomorphology (1), Military affairs (1)
		Ming Dynasty	17	Direction (4), Geomorphology (3), Surnames (3), Religion (3), Military affairs (1), Transportation (2), Hydrology (1)
		From the Qing Dynasty to the modern era	12	Geomorphology (4), Direction (3), Hydrology (1), Military affairs (1), Economic activities (1), Transportation (1), Religion (1)
	Mountainous area (left)	Before the Yuan Dynasty	19	Surnames (5), Hydrology (4), Geomorphology (3), Military affairs (2), Economic activities (2), Religion (2), Direction (1)
		Yuan Dynasty	1	Geomorphology (1)
		Ming Dynasty	11	Surnames (4), Transportation (3), Direction (1), Geomorphology (1), Hydrology (1), Flora and fauna (1)
		From the Qing Dynasty to the modern era	18	Direction (4), Geomorphology (4), Surnames (3), Hydrology (3), Economic activities (2), Transportation (2)
Climate transition zone	North side (dry/nomadic)	Before the Yuan Dynasty	1	Hydrology (1)
		Ming Dynasty	3	Geomorphology (2), Transportation (1)
		From the Qing Dynasty to the modern era	18	Economic activities (5), Hydrology (2), Flora and fauna (2), Surnames (2), Architecture (2), Religion (2), Direction (1), Geomorphology (1), Military affairs (1)
	South side (wet/agricultural)	Before the Yuan Dynasty	11	Military affairs (5), Surnames (3), Hydrology (2), Religion (1)
		Ming Dynasty	15	Military affairs (5), Economic activities (3), Geomorphology (2), Surnames (2), Direction (1), Flora and fauna (1), Transportation (1)
		From the Qing Dynasty to the modern era	14	Military affairs (4), Surnames (3), Flora and fauna (2), Transportation (2), Direction (1), Geomorphology (1), Hydrology (1)

Note: This study focuses on the geographical boundaries of Hebei Province and employs a 15-km buffer zone to analyze toponymic changes. The data integrate township-level place names from the pre-Yuan period to modern times, mapped against the Taihang Mountains, the Ming Great Wall boundaries, and municipal base maps.

As shown in Table 3, prior to the Yuan Dynasty, place names in the plain regions reflected a highly complex social structure, with frequent occurrences of categories such as religion, direction, transportation, geomorphology, and military affairs. However, the Yuan Dynasty experienced a dual disruption in both the quantity and diversity of place names. Although partial recovery occurred during the Ming and Qing periods—particularly through the reappearance of directional terms and surnames—place names related to military affairs and economic activities did not regain their pre-Yuan prominence. This evolutionary trajectory suggests that plain regions were more susceptible to severe social upheavals, with settlement functions shifting from complexity toward simplification.

In contrast, the evolution of place names in the mountainous transition zone exhibited strong continuity and structural stability, with surname-based place names consistently maintaining a high proportion. Although the Yuan Dynasty saw a sharp decline in overall numbers, leaving only limited natural elements such as geomorphology, the Ming Dynasty experienced a notable increase in transportation-related place names. This trend reflects the enhanced role of mountainous areas in strategic transportation routes and military defense. From the Qing Dynasty to the modern era, place-name types became increasingly diverse, indicating a gradual rebalancing between human and natural functions and an overall tendency toward restoring the pre-Yuan structural pattern.

An analysis of place names in the climate transition zone reveals distinct evolutionary trajectories. The northern sector exhibits a clear phase shift: before the Yuan Dynasty, place names were dominated by hydrological terms, while the Ming period introduced geomorphological and transportation-related categories. From the Qing Dynasty onward, economic activity-related names became dominant, accounting for 27.7% of the total. This shift was accompanied by substantial growth in architectural and surname-based names, reflecting structural transformations driven by migration during the Qing period [44].

By contrast, the southern sector exhibits stronger path dependence. Place names related to military activity consistently accounted for 30%–45% across dynasties, underscoring the long-standing role of the Great Wall as a military buffer zone. The continued increase in surname-based names further reflects the gradual transition from military settlements to civilian communities, highlighting the underlying process of civilianization.

#### Analysis of factors influencing toponymic evolution.

To systematically examine the mechanisms underlying the spatial differentiation of township place names in Hebei Province, a categorized analytical framework is adopted. For natural environment place names, geospatial detectors are combined with spatial autocorrelation models to quantify single-factor explanatory power, interaction effects, and spatial clustering characteristics. For cultural place names, the analysis focuses on four key dimensions—ethnicity, migration, military, and economy—using comprehensive statistical analysis and historical verification to clarify the driving mechanisms of cultural factors. In addition, the constraining influence of the natural environment on cultural place names is examined by analyzing their interrelationships.

#### Factors influencing natural environment place names.

Based on the study by Lu et al. [37], the factors influencing the spatial distribution of natural environment place names were classified into elevation (X1), slope (X2), distance to rivers (X3), and vegetation coverage (X4), followed by Geodetector analysis.

The results are presented in Table 4.

**Table 4:** The factor detector results.

Factor	X1	X2	X3	X4
*q* (Geomorphology)	0.516	0.636	0.516	0.642
*q* (Hydrology)	0.046	0.342	0.670	0.428
*q* (Flora and fauna)	0.036	0.080	0.110	0.113

An analysis of Table 4 indicates that the spatial distribution of geomorphological place names is primarily controlled by slope gradient and vegetation coverage. Among these factors, vegetation coverage (X4) exhibits the highest *q*-value (0.642), followed closely by slope gradient (X2) with a *q*-value of 0.636, both substantially exceeding the explanatory power of the remaining factors. Together, these two variables explain more than 63% of the spatial differentiation of geomorphological place names, reflecting their strong environmental adaptability to the gentle slopes of the eastern foothills of the Taihang Mountains and the southern foothills of the Yanshan Mountains.

Hydrological place names display the strongest dependence on distance to rivers, with a *q*-value of 0.670, far exceeding that of other factors. This result highlights a pronounced linear clustering pattern along the tributaries of the Haihe River, indicating that the distribution of water systems constitutes the primary constraint governing the spatial organization of hydrological place names. In contrast, natural environmental factors exert relatively weak explanatory power on flora- and fauna-related place names, with all *q*-values below 0.12. Only vegetation coverage (*q* = 0.113) and distance to rivers (*q* = 0.110) show weak correlations, while elevation (X1) demonstrates minimal explanatory power (*q* = 0.036).

To further examine the synergistic effects among different factors influencing the spatial distribution of place names, the interaction detector of the Geodetector model was employed to quantify the explanatory power of pairwise factor combinations, as shown in Table 5.

**Table 5:** The interaction detection results of factors influencing natural environment place names.

	X1	X2	X3	X4
**X1**	0.317			
**X2**	0.939*	0.195		
**X3**	0.952*	0.563	0.446	
**X4**	0.885	0.953*	0.952	0.723

Note: * indicates nonlinear enhancement.

The interaction analysis in Table 5 shows that vegetation coverage has the strongest single-factor explanatory power for natural environment place names (72.3%). More importantly, two-factor interactions exhibit significant synergistic effects, with most factor combinations showing higher explanatory power than any single factor and demonstrating nonlinear enhancement characteristics. In particular, the interaction between slope gradient and vegetation coverage reaches the highest explanatory power (95.3%), indicating that their coupled influence substantially strengthens the explanation of the spatial distribution of place names. Similarly, the interactions between elevation and distance to rivers, as well as between distance to rivers and vegetation coverage, also show high explanatory power (95.2%).

#### Spatial autocorrelation analysis.

As shown in Table 6, the clustering effects of terrain and vegetation factors exhibit clear temporal variation. The Moran’s *I* indices for elevation, slope, and vegetation coverage display highly significant positive spatial autocorrelation, reaching peak values (*P* < 0.001) during the Yuan Dynasty. Notably, elevation has a Moran’s *I* of 0.706358 (*Z* = 8.59), while slope and vegetation coverage reach 0.299 and 0.302, respectively. These results support the interpretation that military conflicts during the Yuan Dynasty compelled populations to adopt defensive settlement strategies, resulting in clustering within high-altitude and topographically complex areas characterized as easy to defend but difficult to attack [45,46].

**Table 6:** The spatial autocorrelation (Moran’s *I*) results.

Variable	Period	Moran’s *I*	*Z-*value	*P*-value
Elevation	Before the Yuan Dynasty	0.163	0.474	0.635
	Yuan Dynasty	0.706	8.588	0.000
	Ming Dynasty	0.124	0.426	0.670
	From the Qing Dynasty to the modern era	0.0418	0.010	0.921
Slope	Before the Yuan Dynasty	0.152	0.447	0.655
	Yuan Dynasty	0.299	3.929	0.000
	Ming Dynasty	0.007	0.028	0.977
	From the Qing Dynasty to the modern era	0.009	0.026	0.979
Vegetation coverage	Before the Yuan Dynasty	0.002	0.184	0.854
	Yuan Dynasty	0.302	3.775	0.000
	Ming Dynasty	0.005	0.024	0.981
	From the Qing Dynasty to the modern era	0.006	0.022	0.982
Distance to rivers	Before the Yuan Dynasty	0.349	1.005	0.315
	Yuan Dynasty	0.368	4.708	0.000
	Ming Dynasty	0.280	15.818	0.000
	From the Qing Dynasty to the modern era	0.681	30.819	0.000

In subsequent periods, the spatial autocorrelation of these factors weakened markedly or became statistically insignificant, with distributions tending toward randomness. For example, the Moran’s *I* value for elevation during the Ming Dynasty declined sharply to 0.124 (*P* ≈ 0.670), while slope and vegetation coverage indices from the Qing Dynasty to the modern era fell below 0.01, all with *P*-values exceeding 0.9. These results indicate the absence of significant clustering patterns in later periods.

In contrast, hydrological factors were found to follow a distinct evolutionary trajectory. The Moran’s *I value* for distance to rivers remained significantly positive from the Yuan Dynasty onward (*I* = 0.368, *Z* = 4.708). Although minor fluctuations occurred during the Ming Dynasty, the level of significance increased substantially, with the *Z*-value rising from 4.708 in the Yuan Dynasty to 15.818 in the Ming Dynasty, and ultimately peaking from the Qing Dynasty to the modern era (*I* = 0.681, *Z* = 30.819).

Overall, the formation mechanisms of natural environment place names reflect an interaction between environmental constraints and historical adaptive strategies. Their spatial distribution is shaped by nonlinear interactions among multiple factors: geomorphological place names depend on the combined effects of slope and vegetation coverage; hydrological place names exhibit pronounced linear clustering along river systems; and flora- and fauna-related place names show relatively weak environmental dependence. From a temporal perspective, warfare during the Yuan Dynasty encouraged clustering in defensible, high-altitude terrain, whereas from the Ming and Qing Dynasties onward, place name distribution increasingly reflected dependence on water resources. This shift illustrates a broader transformation in human–land relationships, from terrain-based adaptation to resource-oriented utilization.

#### Analysis of factors influencing cultural place names.

**(1) Place names in ethnic settlement areas**

**Fig 6:**
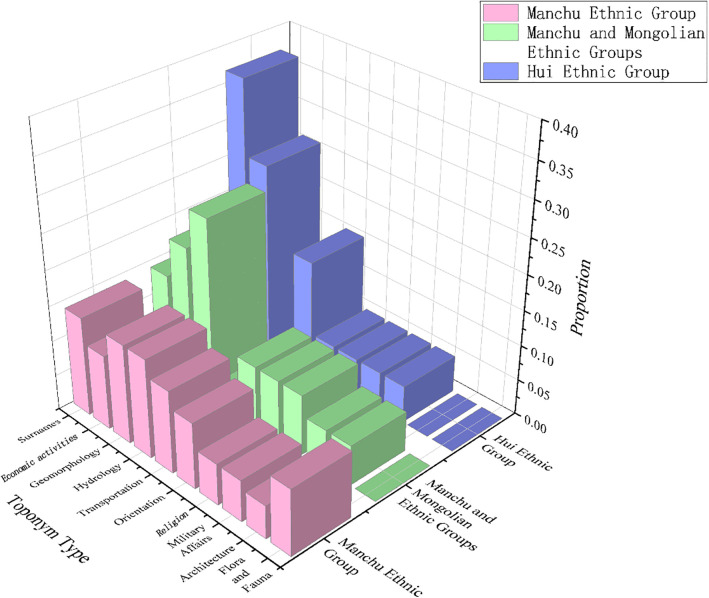
The proportions of place-name types by ethnic groups. Note: Data were sourced from the statistics of ethnic counties or townships.

Quantitative analysis of place name characteristics in ethnic counties and townships (Fig 6) reveals substantial variation across different ethnic communities. In Manchu-populated areas, geomorphological and hydrological place names dominate, with transportation-related terms also accounting for a considerable share, reflecting a strong dependence on the natural environment and mobility. In Manchu–Mongolian cohabitation areas, geomorphological place names account for 25.7% of the total, while economic activity-related terms comprise 20%. This pattern indicates that settlement naming remains closely associated with geographical features and continued reliance on natural environments such as mountainous terrain and river valleys. The relatively high proportion of economic terms highlights the importance of markets, pastoral activities, and trade routes in local spatial organization, reflecting the deep integration of agro-pastoral systems and trade networks in these regions.

In Hui-populated areas, surname-based place names constitute 36.8% of the total, accompanied by a relatively high proportion of economic activity-related terms. The predominance of surnames reflects a strong clan-based spatial structure, in which family names define settlement units and indicate a social organization centered on kinship and religious communities [47]. Furthermore, the prominence of economic terms underscores the importance of markets, handicrafts, and trade networks, with commercial traditions clearly embedded in the toponymic landscape, demonstrating the strong commercial culture and market-oriented economic practices of the Hui community [48].

**(2) Population migration and the toponymic landscape**

The Hongtong Mass Migration of the Ming Dynasty in Shanxi represents a large-scale and far-reaching demographic event in the history of Hebei [49], providing key empirical evidence for understanding how migration has shaped toponymic patterns. A statistical analysis of Table 7 reveals clear typological characteristics among migration-related place names. Names combining “surname + common name” dominate, accounting for 42% of the total, indicating that kinship-based clan organization served as the primary mechanism for reconstructing immigrant societies [14]. Hydrological place names rank second, reflecting settlement concentration along rivers, branch canals, and irrigation systems, and documenting the migration routes along river valleys as well as the water-dependent development of villages [48].

**Table 7:** The proportions of different types of migration-related place names.

Place name type	Quantity	Proportion (%)
Geomorphology	2	4
Hydrology	9	18
Flora and fauna	2	4
Surnames	21	42
Military affairs	2	4
Economic activities	1	2
Architecture	4	8
Transportation	3	6
Religion	2	4
Direction	4	8
Total	50	100

Note: Data were compiled from township place names with immigrant backgrounds.

**(3) Influence of economic activities on the types and spatial distribution of place names**

**Fig 7:**
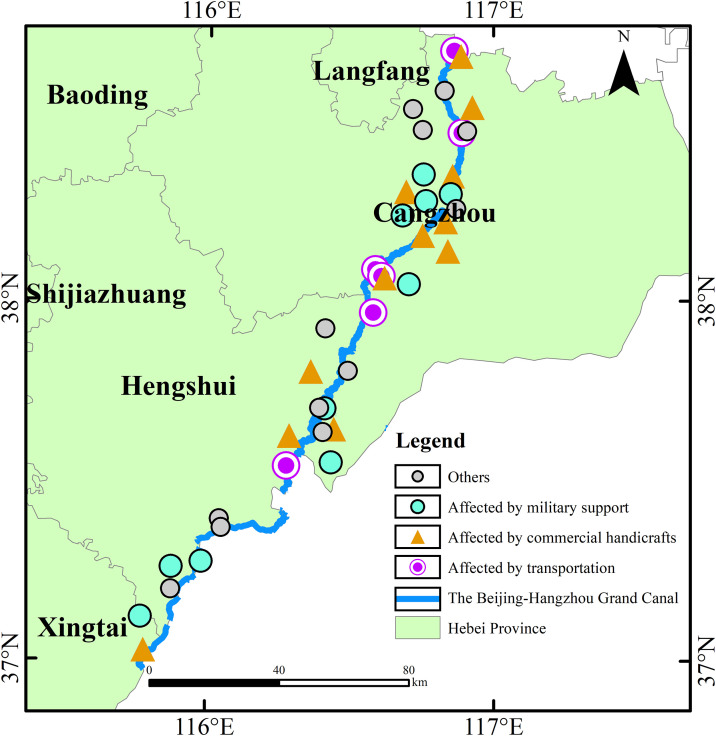
The distribution of township place names near the Beijing–Hangzhou Grand Canal. Note: This figure was created using map data from Tianditu, available at http://www.tianditu.gov.cn/. The data for the Beijing-Hangzhou Grand Canal is sourced from OpenStreetMap (https://www.openstreetmap.net.cn/) and is freely available under the Open Database License (ODbL). Canal features are redrawn from the original data for illustrative purposes only.

During the Ming and Qing Dynasties, the Beijing–Hangzhou Grand Canal functioned as the economic lifeline of China, serving both as a grain transportation corridor and a hub for north–south trade, thereby constituting a critical economic artery [50,51]. Buffer zone analysis (Fig 7) identifies 41 towns within a 10-km radius of the canal. Commercial and handicraft-related place names account for 29.2% of the total, followed by names related to military affairs (24.4%) and transportation (17%). This uneven distribution reflects varying intensities of economic activity along the canal, with commercial and handicraft sectors exerting the strongest influence. Spatially, three distinct patterns can be identified: transportation-related names cluster near waterways; commercial and handicraft-related names concentrate at medium to longer distances from the canal; and military-related names display a mixed distribution pattern, appearing both near and farther from the canal. Together, these toponymic patterns form a spatial representation of the canal’s integrated grain transport, trade, and service systems.

**(4) Interaction between military function and the evolution of place-name types**

Hebei Province has long stood at the frontier of conflict and integration between Central Plains regimes and northern nomadic groups. Frequent warfare profoundly reshaped the region’s cultural landscape [52]. The Taihang Mountains and the Great Wall together constitute the core military–geographical framework of Hebei, serving as key drivers of military conflict formation, defense system development, and ethnic integration. These factors clearly reflect the evolutionary patterns and cultural meanings of military-related place names in the province.

As shown in Table 8, a 10-km buffer zone centered on the Eight Passes of the Taihang Mountains was constructed, and military-related place names within this area were extracted and analyzed using the spatial alignment of the Great Wall and county boundaries as reference points. The results indicate that military place names along the Taihang Mountains and the Great Wall were relatively scarce prior to the Ming Dynasty. The Ming Dynasty marks the peak period for the formation of military-related place names in Hebei, with frequent use of terms such as *Guan* (fortress) and *Ying* (camp). This pattern reflects the dominance of military defense functions in place-name formation during this period. After the Qing Dynasty, as political territories inside and outside the Great Wall were unified, the military connotations of place names gradually weakened, signaling a historical shift in human–land relations from military confrontation to peaceful governance.

**Table 8:** The number and types of military-related township place names near the Great Wall and the Eight Passes of the Taihang Mountains in Hebei Province by dynasty.

Dynasty	Number of place names	Place names
Han	1	Flowing Stream
Sui and Tang	2	Tianchang, Dongbaima
Song	2	Nanmazhuang, Mujicun
Yuan	1	Nam Suu Lin
Ming	10	Sunzhuang, Nantun, Liujiazhuang, Zijingguan, Xixiaying, Jianchangying, Wuzhong'an, Shanhaiguan, Zhenningbao, Chongyukou
Unmixed	1	Badao River
Modern era	4	Wulitai, Yiyuankou, Liujiaying, Yanheyin
Unknown	3	Xixu, Huolu, Daomaguan
Total	24	

In summary, the formation of cultural place names represents the spatial codification of social structures. This process operates through a four-dimensional integration of “subject–institution–activity–narrative.” Within specific institutional frameworks—such as clan systems, grain transportation networks, and military defense systems—diverse social actors (clans, merchant groups, and state authorities) translate identity, functional roles, and collective memory into a toponymic system through spatial practices including migration, trade, and garrisoning. Through this mechanism, fluid social relations are transformed into stable spatial texts, establishing place names as institutionalized carriers of geopolitical order, economic organization, and cultural identity.

## Discussion

The spatiotemporal evolution of toponymic cultural landscapes is fundamentally the result of long-term interactions between human and natural systems, a process that can be systematically interpreted through the analytical framework of the Yearbook School [53]. This theory divides historical evolution into relatively stable physical geographical backgrounds, gradually changing socioeconomic structures, and sudden historical events, and can effectively reveal the multi-level driving mechanisms of toponyms in transition zones.

Hebei Province is located in a dual transition zone of topography and climate, and the evolution of its township-level place names is highly consistent with this analytical framework. Over the long term, the landform and precipitation gradients along the Taihang Mountains–Great Wall line have remained relatively stable, forming the natural foundation of the toponymic pattern. The differentiation characterized by stable mountain toponyms and easily altered plain toponyms has persisted over time. Over the medium term, beginning in the Ming and Qing dynasties, the area along the Great Wall gradually transformed from a military defense zone into a transition zone where agriculture and commerce coexisted. This is reflected in the large emergence of military toponyms such as Guan (pass), Ying (garrison), and Bao (fort) during the Ming Dynasty, which were gradually replaced by surname-based and economic toponyms after the Qing Dynasty (e.g., the evolution from Zhangjiaying to Zhanggezhuang). This “military-to-civilian” transition reflects improvements in productivity, population growth, and the shift from military-oriented land control to sedentary agricultural development, driving toponymic evolution from natural environment dominance toward cultural dominance. In the short term, events such as dynastic transitions, wars, and the establishment of military garrisons triggered abrupt changes, including discontinuous fluctuations of place names during the Yuan Dynasty and the concentrated emergence of military toponyms in the Ming Dynasty. Furthermore, immigration in the Ming Dynasty and the construction of the Great Wall respectively contributed to the rapid increase of surname-based toponyms in the plains and the zonal clustering of military toponyms along the Great Wall.

In summary, the evolution of toponyms in transition zones results from the combined effects of long-term natural environmental constraints, medium-term socioeconomic transformation, and short-term political events. This process reveals the hierarchical structure and historical accumulation of human–nature coupling.

Compared with the study by Wang et al. [23] on county-level administrative place names in the eastern plains of China, the evolution of place names in plain areas demonstrates a relatively continuous and stable natural–cultural transition, whereas transition zones exhibit stronger characteristics of rupture and reorganization (such as the leapfrogging development of economic place names on the northern side and the transformation from military to civilian functions on the southern side). This indicates that geographical transition zones are not only areas of natural gradient changes but also response areas sensitive to historical events, with higher discontinuity and mutability in toponymic evolution.

On the other hand, toponymic research in Western countries is often constrained by relatively short historical records, and tends to focus on contemporary spatial distribution, power symbolization, and identity recognition [12], making it difficult to conduct long-term dynamic analyses spanning several centuries. In contrast, this study relies on China’s rich local chronicles and long historical continuity, enabling a cross-dynastic spatiotemporal coupling analysis from the pre-Yuan period to the Qing Dynasty and modern times, thereby revealing the evolutionary trajectory and transformation nodes of the toponymic landscape within long-term historical change.

Based on the findings, region-specific recommendations are proposed for the protection of toponymic cultural heritage in Hebei Province. In transition zones, priority should be given to the protection of landform-related place names along the Taihang Mountains (e.g., Jingxing, Fuping) and military place names along the Great Wall (e.g., Zijingguan, Shanhaiguan). For settlements that have undergone a transformation from military to civilian functions, cultural and tourism development should be integrated to better interpret the historical narratives embedded in place names and strengthen the transition of collective memory. In non-transition zones such as the central–southern plains, focus should be placed on protecting hydrological, commercial, and clan-based settlement place names (e.g., Botou, Matou), while strictly controlling dehistoricized renaming practices during urbanization processes (e.g., changing Gucheng to Xincheng) to maintain cultural continuity. It is suggested that civil affairs and cultural tourism authorities conduct a systematic census of toponymic changes every five years, and that the proposed protection strategies be extended to other similar regions in North China.

In addition to the aforementioned findings, this study was characterized by several limitations. The limitations of etymological interpretation may affect the accuracy of flora- and fauna-related place name identification [54].Furthermore the research scope was confined to the township level and did not include village-level place names. Moreover, the absence of historical records in some townships restricted the dynamic reconstruction of long-term evolutionary processes. Finally, the study was limited to Hebei Province, and the cross-regional applicability of the findings remains to be validated. In future research, multi-scale toponym databases can be integrated, and dynamic simulations can be carried out by combining local chronicles and spatial big data, along with cross-regional comparative studies. Based on regional case studies, a theoretical framework for toponymic evolution suited to China’s national context should be constructed to provide stronger academic support for the protection, revitalization, inheritance, and development of regional toponymic culture. Further attention should also be paid to micro-level village place names and cross-regional comparative research. The analytical framework of spatiotemporal coupling and transition zones adopted in this study may also be extended to other cultural landscapes, such as traditional villages and agricultural heritage sites.

## Conclusion

Based on data from 1,854 township-level place names in Hebei Province, China, this study comprehensively employed kernel density estimation, the Geodetector model, and spatial autocorrelation methods to reveal the spatiotemporal evolution patterns and driving mechanisms of the toponymic cultural landscape in the study area. Over the study period, natural environment place names contracted from early multi-core agglomerations in the plains toward the mountainous northeast and coastal areas, while the core zones of cultural place names continuously shifted northward from the central–southern plains, reflecting long-term adjustments in regional human–land relationships.

The transition zone between the Taihang Mountains and the Great Wall exhibits distinctive regional characteristics: the southern areas and the eastern foothills of the Taihang Mountains are dominated by military place names, whereas the northern regions and central–southern plains are characterized by economic place names. During the Ming Dynasty, a proliferation of military place names emerged in the Taihang Mountains and Great Wall area; after the Qing Dynasty, these names gradually shifted from military to civilian usage. Their evolution was a result of the superimposed effects of long-term natural foundations, medium-term socioeconomic transitions, and short-term political events.

The innovative perspective of this study lies in treating geographical transition zones as independent analytical units, systematically revealing their distinctive toponymic evolutionary patterns compared with single landform units. Furthermore, this study achieved long-term, multi-factor spatiotemporal coupling analysis, overcoming the limitation of traditional research that emphasizes spatial patterns while neglecting temporal dynamics. Theoretically, it enriches the understanding of human–land relationship evolution in transition zones; practically, it provides a scientific basis for the protection of toponymic cultural heritage in Hebei Province and similar transitional regions across North China.

## Supporting information

S1 Dataset Hebei toponymy. (XLS)

S2 Dataset Influencing factors. (XLS)
